# Methylation and Expression of Mutant *FUS* in Motor Neurons Differentiated From Induced Pluripotent Stem Cells From ALS Patients

**DOI:** 10.3389/fcell.2021.774751

**Published:** 2021-11-19

**Authors:** T. Hartung, M. Rhein, N. Kalmbach, N. Thau-Habermann, M. Naujock, L. Müschen, H. Frieling, J. Sterneckert, A. Hermann, F. Wegner, S. Petri

**Affiliations:** ^1^ Department of Neurology, Hannover Medical School, Hannover, Germany; ^2^ Department of Neurology, Charité–Universitätsmedizin Berlin, Berlin, Germany; ^3^ Department of Psychiatry, Social Psychiatry and Psychotherapy, Hanover Medical School, Hanover, Germany; ^4^ Evotec International GmbH, Göttingen, Germany; ^5^ Center for Regenerative Therapies TU Dresden (CRTD), Technische Universität Dresden, Dresden, Germany; ^6^ Translational Neurodegeneration Section “Albrecht Kossel”, Department of Neurology and Center for Transdisciplinary Neuroscience (CTNR), University Medical Center Rostock, University of Rostock, Rostock, Germany; ^7^ German Center for Neurodegenerative Diseases (DZNE) Rostock/Greifswald, Rostock, Germany

**Keywords:** amyotrophic lateral sclerosis, induced pluripotent stem cells derived motor neurons, *FUS*, methylation, DNA methyltransferases

## Abstract

Amyotrophic lateral sclerosis (ALS) is a rapidly progressive disease leading to degeneration of motor neurons (MNs). Epigenetic modification of gene expression is increasingly recognized as potential disease mechanism. In the present study we generated motor neurons from induced pluripotent stem cells from ALS patients carrying a mutation in the fused in sarcoma gene (FUS) and analyzed expression and promoter methylation of the FUS gene and expression of DNA methyltransferases (*DNMTs*) compared to healthy control cell lines. While mutant *FUS* neural progenitor cells (NPCs) did not show a difference in *FUS* and *DNMT* expression compared to healthy controls, differentiated mutant *FUS* motor neurons showed significantly lower *FUS* expression, higher *DNMT* expression and higher methylation of the proximal *FUS* gene promoter. Immunofluorescence revealed perceived proximity of cytoplasmic *FUS* aggregates in ALS MNs together with 5-methylcytosin (5-mC). Targeting disturbed methylation in ALS may therefore restore transcriptional alterations and represent a novel therapeutic strategy.

## Introduction

Amyotrophic lateral sclerosis (ALS) is a neurodegenerative disease of the motor system leading to death after 3–5 years from symptom onset, mainly due to respiratory insufficiency (Talbot, 2009). During the course of disease, patients typically show symptoms of upper motor neuron dysfunction (spasticity, increased deep tendon reflexes) and lower motor dysfunction (muscle wasting, weakness and fasciculations). While 90% of cases are sporadic, 10% show familial clustering. To date 25 genetic loci associated with familial ALS (fALS) have been identified, among others chromosome nine open reading frame 72 (C9orf72), which accounts for about 45% of fALS cases, followed by Superoxid Dismutase-1 (SOD1), which accounts for about 20% and Transactive response DNA binding protein 43 kDa (TDP-43) and fused in sarcoma gene (FUS), which each account for approximately 5% (Brown and Al-Chalabi, 2017; van Es et al., 2017). Sporadic ALS is clinically indistinguishable from the familial form and most probably caused by complex gene-environment interactions. A key neuropathological feature of ALS are cytoplasmic protein aggregates associated with progressive neuronal loss, due to downstream effects of loss of function or toxic gain of function of the protein. Protein aggregate pathology shows a characteristic spreading pattern across specific brain regions with disease progression, suggesting a “prion-like” spreading mechanism (Brettschneider et al., 2013). Up to date this multifactorial process is not fully understood. Excitotoxicity, disturbed RNA transport and splicing, axonal protein transport and mitochondrial function appear to be involved. While this process may be initiated by distinct monogenetic mutations in the 10% familial cases, its precise pathophysiology in the 90% sporadic cases is still unclear. In recent years, epigenetic and post-transcriptional modifications are getting more attention in this context.

Epigenetics encompasses all mechanisms, which regulate gene expression without changing genetic information. These mechanisms play an important role in cell differentiation and cell identity, but also in disease development. The three most studied epigenetic mechanisms are histone modification, which regulates the chromatin state; non-coding RNAs, which block messenger RNA, and DNA methylation, which can directly inhibit transcription (Bonasio et al., 2010). DNA methylation occurs mainly at the 5^th^ atom of cytosines (5-mC) followed by a guanine termed CpG site. The human genome approximately contains 29 million of these CpG sites, which split up into regions with high density (so-called CpG islands) and low CpG regions, located close to the transcription start site, i.e., the promoter of a gene. DNA methylation at these sites is considered to interfere with gene expression whereby differences have been shown according to CpG density (Hartung et al., 2012). The main enzymes regulating DNA methylation are DNA methyltransferase 1 (*DNMT1*) which is involved in maintaining methylation patterns during replication and *DNMT3a* and *DNMT3b*, which are involved in *de novo* methylation during the early embryonic phase and cell differentiation (Hermann et al., 2004). *DNMTs* are highly expressed in post-mitotic neurons suggesting an important functional role in the nervous system and neuronal disorders (Feng and Fan, 2009). Mutations in the *DNMT1* gene in mice are lethal after approximately 11 days and knock-out of *DNMT3a/b* lead to severe defects in embryogenesis (Li et al., 1992; Okano et al., 1999). Deletion of *DNMT1 in vitro* in neural stem cells results in decrease of newly generated mature neurons during adult neurogenesis in the dentate gyrus of the hippocampus (Noguchi et al., 2015). *DMNT3a* remains active in adult post-mitotic neurons and when deleted, mice develop fewer motor neurons and exhibit motor deficits suggesting an important role in motor neuron development and movement (Nguyen et al., 2007). During tumorigenesis human cancer cells develop promoter CpG-island hypermethylation and lose CpG methylation in non-CpG-island promoters resulting in cancer-specific methylation patterns. Although disturbed DNA methylation is most prominent in Cancer cells, distinct DNA methylation profiles can be found in other diseases as well (Fernandez et al., 2012). Besides *DNMTs* regulating transcription, the RNA transferase *DNMT2* got more attention recently because it may be a key player in post-transcriptional regulation under stress conditions (Schaefer et al., 2010). Several studies have shown a correlation between changes in methylation and neurodegeneration. For example, apoptosis of cultured neurons is correlated with higher expression of *DNMTs*. While enforced expression of *DNMTs* was shown to induce apoptosis in cultured neurons, *DNMT* inhibition prevented apoptotic cell death (Chestnut et al., 2011; Martin and Wong, 2013). In a stroke animal model, reduced levels of *DNMT1* were associated with protection of post-mitotic neurons against ischemic brain injury (Endres et al., 2001). Inducing neuronal apoptosis by sciatic nerve avulsion in mice resulted in increased DNA methylation and treatment with the *DNMT1* inhibitor RG108 prevented motor neuron loss (Chestnut et al., 2011). Discovering epigenetic mechanisms in ALS could be relevant for new disease target identification and therapies. Epigenetic research in ALS initially concentrated on sporadic ALS cases assuming that epigenetic modifications may lead to differential gene expression profiles driving neurodegeneration and leading to the ALS phenotype and that initiation of epigenetic modifications may be influenced by exposure to specific environmental factors with subsequent impact on disease onset and progression. Motor neuron apoptosis in sporadic ALS is associated with an upregulation of *DNMT1* and *DNMT3a* and increased 5-mC (Chestnut et al., 2011). An immunohistochemistry study of neurons of the post-mortem motor cortex of sporadic ALS patients showed increased immunoreactivity of *DNMT1*, *DNMT3A* and 5-mC compared to controls (Chestnut et al., 2011). Genome-wide analysis of DNA methylation in post-mortem brains of sporadic ALS patients identified differentially methylated regions in possible new candidate genes involved in calcium homeostasis, neurotransmission and oxidative stress (Morahan et al., 2009). Global methylation analysis of post-mortem spinal cord identified 112 genes related to immune and inflammation response being hypo- or hypermethylated and corresponding up- or downregulation in sporadic ALS patients (Figueroa-Romero et al., 2012).

Few studies regarding methylation changes have focused on familial ALS. There is evidence for different epigenetic modifications regulating expression of ALS-causing genes (Jimenez-Pacheco et al., 2017). While global methylation measured in blood from patients with *SOD1* mutations was increased compared to controls, no gene-specific changes were detected. Recently a number of DNA methylation studies reported promoter hypermethylation of the *C9orf72* repeat expansion of ALS patients and corresponding mRNA down-regulation (Xi et al., 2013; Liu et al., 2014; Russ et al., 2015; Gijselinck et al., 2016). Furthermore, Shi et al. (2018) could show that suppression of *C9orf72* transcription by antisense oligonucleotide-mediated knockdown reduced survival of cultured motor neurons whereas restoring *C9orf72* transcription rescued motor neuron survival. These studies demonstrate that DNA methylation profiles could potentially act as epigenetic biomarkers in ALS and even be a therapeutic target. In the present study, we have analyzed for the first time site-specific promoter methylation and expression of *FUS* and *DNMTs* in ALS patient derived motor neurons with mutated *FUS*, aiming to contribute to increased understanding of the role of epigenetics in ALS and potential novel therapeutic approaches.

## Materials and Methods

### Differentiation of Motor Neurons

All experiments were performed in compliance with the Helsinki convention and approved by the ethical committees of Hannover. ALS patient-derived induced pluripotent stem cells (iPSC) and healthy control-derived iPSC were generated and characterized including karyo- and genotyping as described previously (Reinhardt et al., 2013; Naujock et al., 2014; Japtok et al., 2015; Naumann et al., 2018). Neural progenitor cells (NPCs), from three healthy control cell lines and three ALS cell lines, carrying a mutation in the nuclear localization signal (NLS) in exon 15 of the *FUS* gene (either the missense mutation R521L (c.1562G > T, p.Arg521Leu) with a G to T transition leading to replacement of arginine by leucine or the missense mutation R521C (c.1561C > T, p.Arg521Cys) with a C to T transition leading to replacement of arginine by cysteine) were differentiated into motor neurons using an established protocol (Naujock et al., 2014). Characteristics of patients are summarized in Supplementary Table S1. According to this protocol, cells express neuronal- and MN-specific markers within less than 3 weeks and demonstrate neuronal function in calcium imaging and patch-clamp analysis after 30 days. NPCs were expanded and replated 2 times when wells were 80% confluent in 1:10 dilution on Matrigel-coated dishes. NPCs of three different wells per cell line were collected, counted in 4 × 16 squares of a Neubauer counting chamber and frozen for DNA and RNA isolation, resulting in three replicates for each cell line. Cells of the other three wells were replated for further differentiation. On day 25 cells were split in equal ratio on laminin coated six well plates and Falcon^®^™ 8-well culture slides. On day 40 cells of laminin coated six well plates were collected for DNA and RNA isolation, cells on Falcon^®^™ 8-well culture slides were used for immunofluorescence staining. For DNA and RNA isolation cells from four of 12 wells were combined resulting in 3MN replicates for each cell line.

### Immunofluorescence

For immunofluorescence staining MNs were fixed with 4% formaldehyde for 20 min at room temperature, washed three times with phosphate-buffered saline, and blocked for 60 min in blocking buffer (5% goat serum, 1% bovine serum albumin, and 0.3% Triton X-100). Primary antibodies (mouse monoclonal anti-*TUJ1*, 1:500, Abcam; rabbit polyclonal anti-*ISLET1*, 1:500, Abcam; rabbit polyclonal anti-*MAP2*, 1:500, Abcam; mouse monoclonal anti-*SMI32*, 1:500, Abcam; rabbit polyclonal anti-*FUS*, 1:100, Abcam; mouse monoclonal anti-5-methylcytosine, 1:200, Millipore) were incubated overnight at 4°C. After additional washing steps, secondary antibodies (Alexa Fluor 488 or 555 goat anti-mouse, and goat anti-rabbit 1:1000 or 1:200, respectively) were applied for 2 h at room temperature. DAPI counterstaining in mounting solution was applied for 20 min at room temperature. For immunofluorescence staining for 5-methylcytosin cells were pretreated with ice-cold-methanol for 10 min at −20°C followed by treatment with 2N HCL for 30 min at 37°C to denature the DNA according to the ICC protocol ab214727 from Abcam concerning prior antibody incubation. The mouse monoclonal anti-5-methylcytosine antibody applied detects methylated cytosines in DNA and RNA. Visualization was done by fluorescence microscopy (BX61; Olympus). Images were taken with an Olympus DP72 camera. Fluorophores and filters were chosen with minimal possible overlap in order to minimize crosstalk. Alexa Fluor 488 is a fluorescent compound with an excitation peak at 499 nm and an emission peak at 520 nm, Alexa Fluor 555 with an excitation peak at 553 nm and an emission peak at 568 nm. Filters applied for visualizing DAPI had a center wavelength of 350 nm and bandwidth of 50 nm (350/50), accordingly filters for visualizing Alexa Fluor 488; 485/10 and for Alexa Fluor 555; 525/25. Additionally, single stainings for *FUS* (Alexa Fluor 488), and 5mC (Alexa Fluor 555) were performed in order to further demonstrate the observed co-localisation. The image-analysis software CellSense (Olympus) was used for further evaluation. Cell counts were performed on five random visual fields twice for each cell line. Cells staining positive for *TUJ1* and *ISLET1* or *MAP2* and *SMI32* were determined as MNs. For quantification of nuclear versus cytoplasmic *FUS* aggregates, cells with nuclear-only and cells with nuclear and cytoplasmic *FUS* staining were counted on five random visual fields.

### DNA and RNA Isolation and cDNA Synthesis

DNA and RNA were isolated from NPCs and MNs with peqGOLD TriFast according to manufacturer’s protocol. DNA and RNA concentrations were measured with a NanoDrop spectrophotometer (VWR, Radnor, United States). A total of 250 ng of RNA was used for cDNA synthesis with the QuantiTect Reverse Transcription Kit from Qiagen according to the manufacturer’s protocol.

### Expression Analyses by Quantitative Real-Time Polymerase Chain Reaction

The final quantitative real-time PCR (qRT-PCR) experiments were executed with 2.5 ng of cDNA synthesized from 250 ng of total RNA with 1.75 mM forward/reverse primer, and Power SYBR-Green PCR Master Mix (Life Technologies). Primer design was carried out with NCBI Primer blast and NetPrimer. We tested 37 primer pairs including four housekeeping genes as reference. Primers applied are listed in Supplementary Table S2. The StepOnePlus Instrument (Applied Biosystems) was used for the following cycles: 95°C/10 min and 40 cycles of 95°C/15 s and 60°C/1 min and one cycle of 95°C/15 s, 60°C/1 min and 95°C/15 s. The expression fold change was calculated via the comparative Ct method as previously described (Livak and Schmittgen, 2001). The data is presented as fold change in gene expression normalized to an endogenous reference gene; Glyceraldehyde 3-phosphate dehydrogenase (*GAPDH*) or Peptidylpropyl isomerase A (*PPIA*) and relative to the calibrator respectively e.g., the healthy control when comparing control and ALS samples or NPCs when comparing NPCs with MNs.GraphPad Prism five Software was used for statistical analysis.

### Bisulfite-Sequencing and DNA Methylation Analysis of the *FUS* Promoter

DNA used for bisulfite sequencing was purified with magnetic beads following the MN Nucleomag Blood 200 µL Kit manufacturer’s protocol on a Biomek NXP Automated Workstation (Beckman Coulter, High Wycombe, United Kingdom). Bilsufite conversion was performed using the EpiTect 96 Bisulfit Kit following the manufacturer’s protocol for low concentration solutions. For bisulfite sequencing primer design of the *FUS* promoter region, the Homo sapiens chromosome 16, GRCh38.p13 Primary Assembly Sequence, location 31175138:31196605 for the human *FUS* gene (ENSG00000089280) was taken as a basis. Primers were designed for the region spanning the first exon and 1000 bp upstream, which encompasses a CpG island defined as a region >200 bp with >60% CpGs. The primary sequence of interest was copied in Methyl Primer express. The tool searches for CpG islands and simulates bisulfite modification of DNA in silico. The in silico bisulfite modified DNA was copied in Geneious and primers were manually designed for the forward and reversed strand and tested. PCR products were purified using Agencourt AMPure XP PCR Purification Kit and visualized on a standard 2.0% agarose gel to confirm the product length. Two primer pairs generated a sufficient PCR product and sufficient bisulfite sequencing results, spanning a 467 bp long fragment 1 (F1: TTTGAGAAAAGGTTGGGTAT/R2: ATC​TAT​CTC​CAC​CCC​CAT​AA) and a 211 bp long fragment 2 (F4: TTTTATGGGGGTGGAGATAG/R1: CCT​AAA​AAA​CTA​AAC​AAC​CC) encompassing 35 and 19 CpGs. The PCR program used was the following: 95°C for 15 min, 97°C for 1 min, 15 cycles of 95°C for 30 s, 61°C for 45 s (decrement temperature by 1°C per cycle), 72°C for 2:30 min and 30 cycles 95°C for 30 s, 46°C for 45 s, 68°C for 2:30 min and 65°C for 5 min. Afterwards an additional sequencing PCR was executed applying either F1/R2 for fragment one or F4/R1 for fragment 2. Sequencing PCR program for fragment 1: 96°C for 1 min, 28 cycles of 96°C for 10 s, 50°C for 5 s, 60°C for 4 min. Sequencing PCR program for fragment 2: 96°C for 1 min, 25 cycles of 96°C for 5 s, 60°C for 1:30 min, 50°C for 1:30 min. Probes were sequenced on a Seg HITACHI Applied Biosystems 3500 XL genetic analyzer. Quality control of sequences was performed using Sequence Scanner Software 2 (ABI life technologies, Foster City, CA, United States). Only sequences with a Quality Value of 20+ were included in the analysis. Quantitative methylation measurements for individual cytosine positions after alignment with genomic reference sequences were assessed using the Epigenetic Sequencing Methylation Analysis Software (ESME) (Lewin et al., 2004). A methylation value of a CpG was counted as valid when 95% of reads reported a value for that specific CpG. This resulted in 27 valid CpG sites for fragment 1 and 10 valid CpG sites in fragment 2. Calculation of methylation data occurred through direct sequencing. Analysis through the ESME software package returns relative values of methylation percentage normalized for fluorescence difference. As such, these are values that provide a comparative measure between samples and treatments, which serves the purpose of correlation between methylation and expression data. Global methylation was calculated as the mean of methylation values of all valid CpGs. The mean methylation for a unique CpG in a cell line was calculated by averaging the measurements from all the reads generated during sequencing. Further statistics were performed in SPSS.

### In Silico Transcription Factor Prediction

Using an in silico prediction database consisting of ChipSeq data for binding affinities of transcription factors (www.factorbook.org), we identified seven likely binding factors that could be involved with FUS regulation at significant sites of the proximal promoter. As most transcription factors have a binding motif of 4–10 bases, we used the CG dinucleotide(s) of interest and included four bases on every side.

### Statistical Analysis

GraphPad Prism five Software (GraphPad Software, San Diego, CA, United States, http://www.graphpad.com/) was used for statistical analysis of qRT-PCR data. Statistical inferences were drawn from unpaired *t* test or in case of non-normal distribution from Mann-Whitney test and illustrated as mean ± SEM. In concrete terms final calculations were based on the cycle threshold (CT) normalized against the endogenous housekeeping gene *GAPDH* or *PPIA* [Ct (target) − Ct (reference) = DCt]. Relative levels of gene expression were illustrated on linear scale as 2^−ΔΔCT^means ± standard error of the mean (SEM). Results were regarded as statistically significant with ***p < 0.001, **p < 0.01, *p < 0.05. IBM SPSS Statistics 27 (SPSS) was used for statistical analysis of immunofluorescence and methylation data. Statistical inferences were drawn from unpaired *t* test and regarded as statistically significant with *p* < 0.05.

## Results

### Motor Neuron Differentiation and Specification

**FIGURE 1:**
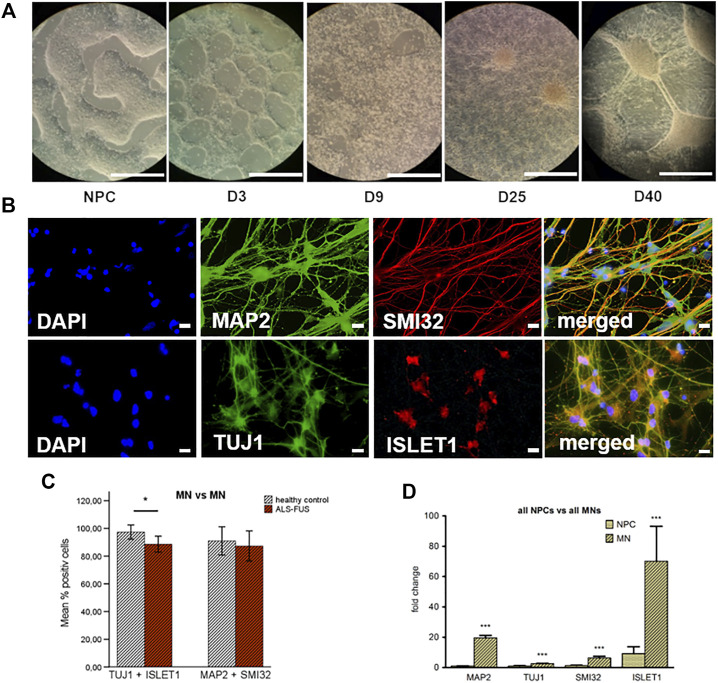
Motor neuron differentiation, **(A)** After differentiation day 9, extensive branching and increased interconnectivity of cells was observed in our colonies, on day 40 mature motor neurons showed complex neurite outgrowth and increased soma areas, **(B)** Immunofluorescence: mature MNs stained positive for neuronal- and MN-specific markers, 88% of all counted cells stained positive for neuronal marker *TUJ1* and MN-associated marker *ISLET1*, 89% of all counted cells stained positive for neuronal marker *MAP2* and MN-associated neurofilament marker *SMI32*, **(C)** Quantitative analysis of the motor neuron populations in neuronal cell culture at D40. Control MNs showed significantly higher count of cells staining positive for *TUJ1* + *ISLET1* than FUS mutant MNs, but no significant difference of cells staining for *MAP2* + *SMI32*, **(D)** Quantitative real-time PCR confirmed immunofluorescence results. MNs expressed significantly more neuronal markers (*TUJ1*/*MAP2*) and MN-specific markers (*SMI32*/*ISLET1*). Graphs show fold change of expression compared to reference gene *GAPDH* or *PPIA*. Statistical inferences were drawn from unpaired *t*-test or in case of non-normal distribution from Mann-Whitney test and illustrated as mean ± SEM. Results were regarded as statistically significant with ***p < 0.001, **p < 0.01, *p < 0.05, *n* = 3 per condition and *n* = 3 per cell line.

IPSC-derived MNs from ALS patients with *FUS* mutations (R521L/R521C) and healthy controls showed extensive branching and increased interconnectivity of cells from day 9 *in vitro*. During further differentiation the cells quickly changed morphology and started to form extensive neuronal networks. On day 40 mature MNs showed complex neurite outgrowth and increased soma areas (Figure 1A). Immunofluorescence indicate differentiation into MN lineage. After 40 days of differentiation, the majority of cells stained positive for neuronal and MN markers, both indicative for mature MN fate (Figure 1B). 88% of all counted cells stained positive for neuronal marker *TUJ1* and MN-associated marker *ISLET1*, 89% of all counted cells stained positive for neuronal marker *MAP2* and MN-associated neurofilament marker *SMI32*, both indicative for efficient MN differentiation. Quantitative analysis of motor neuron populations in control and *FUS* mutant cell lines showed significantly higher MN counts in control cell lines (defined by *TUJ1* and *ISLET1* expression) for *TUJ1*/*ISLET1* (Figure 1C). To further confirm the immunofluorescence observations, NPCs and MNs were analyzed for mRNA expression of neuronal- and MN-specific markers by quantitative RT-PCR. After differentiation, a significant upregulation of neuronal- (*MAP2*/*TUJ1*) and MN-specific markers (*SMI32*/*ISLET1*) was found indicating that MN differentiation occurred in all cell lines (Figure 1D).

### Expression of *FUS* and *DNMTs*

**FIGURE 2:**
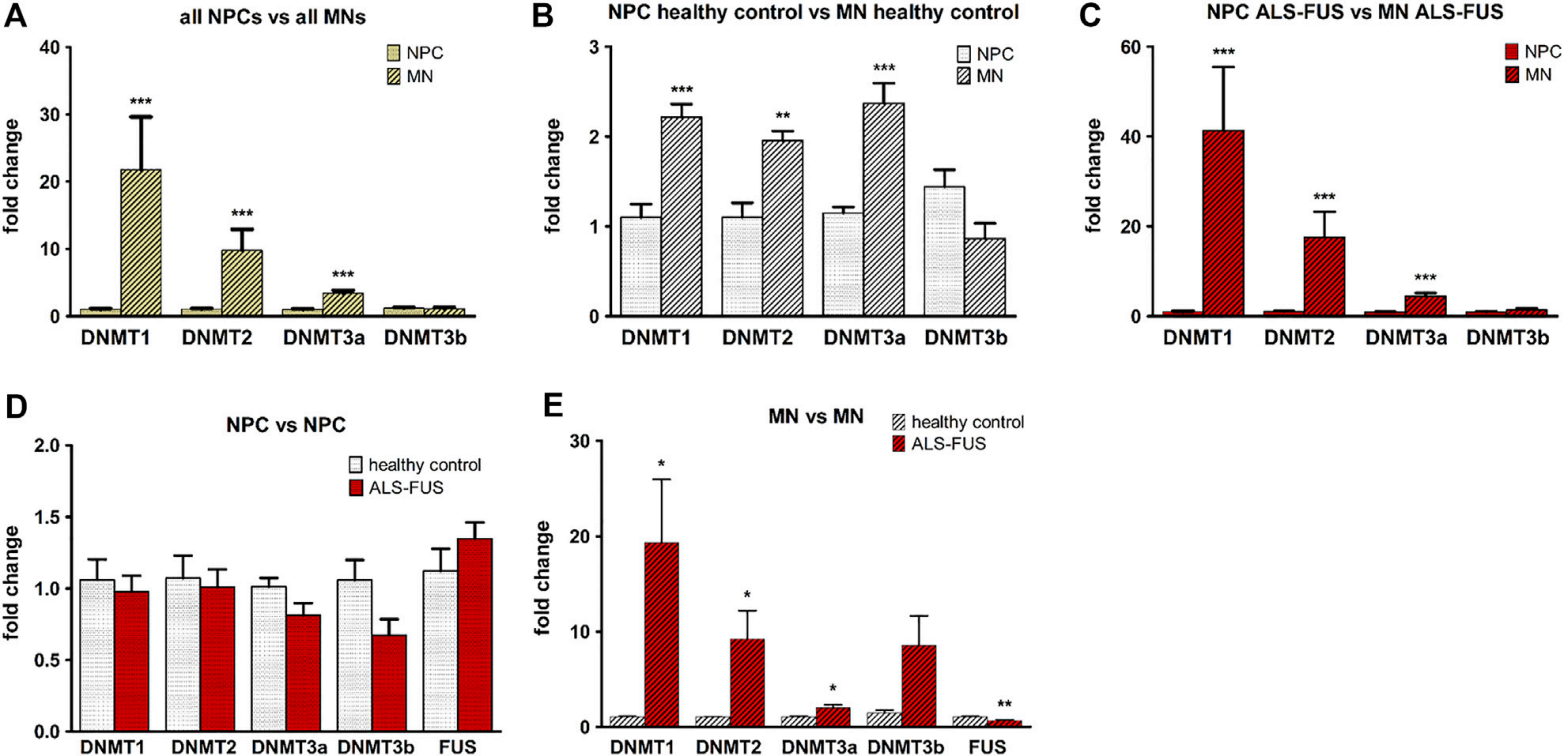
*DNMT* and *FUS* expression. DNA methyltransferase (DNMT) upregulation during MN differentiation, **(A)** Significantly higher expression of DNMT1, DNMT2 and DNMT3a in all MNs compared to all NPCs, **(B)** in MNs of control cell lines compared to NPCs and, **(C)** in MNs of ALS cell lines compared to NPCs. *DNMT* and *FUS* expression in control and ALS cell lines, **(D)** NPCs from control and ALS cell lines showed no significant difference in expression of *DNMT1*, *DNMT2*, *DNMT3a*, *DNMT3b* and *FUS*, **(E)** Motor neurons of ALS cell lines express significantly more *DNMT1/2/3a* and less *FUS*. Graphs show fold change in gene expression normalized to an endogenous reference gene (GAPDH or PPIA) and relative to the calibrator respectively e.g., the healthy control when comparing control and ALS samples or NPCs when comparing NPCs with MNs. Statistical inferences were drawn from unpaired *t*-test or in case of non-normal distribution from Mann-Whitney test and illustrated as mean ± SEM. Results were regarded as statistically significant with ***p < 0.001, **p < 0.01, *p < 0.05, *n* = 3 per condition and *n* = 3 per cell line.

In order to examine DNA methylation and *FUS* expression in relation to ALS pathology we performed qRT-PCR for *DNMT1, 2, 3a, 3b* and *FUS* in NPCs and MNs of ALS cell lines and controls. Independent of the genotype, differentiated MNs at d40 showed significantly higher expression of *DNMT1*, *DNMT2* and *DNMT3a* but not *DNMT3b* than NPCs (Figure 2A). This difference remained significant when looking at control and ALS cell lines during differentiation separately (Figures 2B,C). Furthermore, upregulation of DNMTs during MN differentiation was much higher in ALS cell lines than in control cell lines. While healthy control cell lines show a twofold upregulation of DNMT1, 2 and 3a during differentiation in average, ALS cell lines show 40 fold upregulation of DNMT1 during differentiation from NPCs to MNs, 16 fold upregulation of DNTM2 and three fold upregulation of DNMT3a. Mutant *FUS* and control NPCs showed no significant difference in expression of all five genes (Figure 2D). Mutant *FUS* MNs expressed significantly more *DNMT1/2/3a* and significantly less *FUS* mRNA than control MNs (Figure 2E). Verification of control and mutant *FUS* MN qRT-PCR amplicon sizes via gel electrophoreses and amplification plots are exemplarily shown in Supplementary Figure S1.

### Methylation Analysis

**FIGURE 3:**
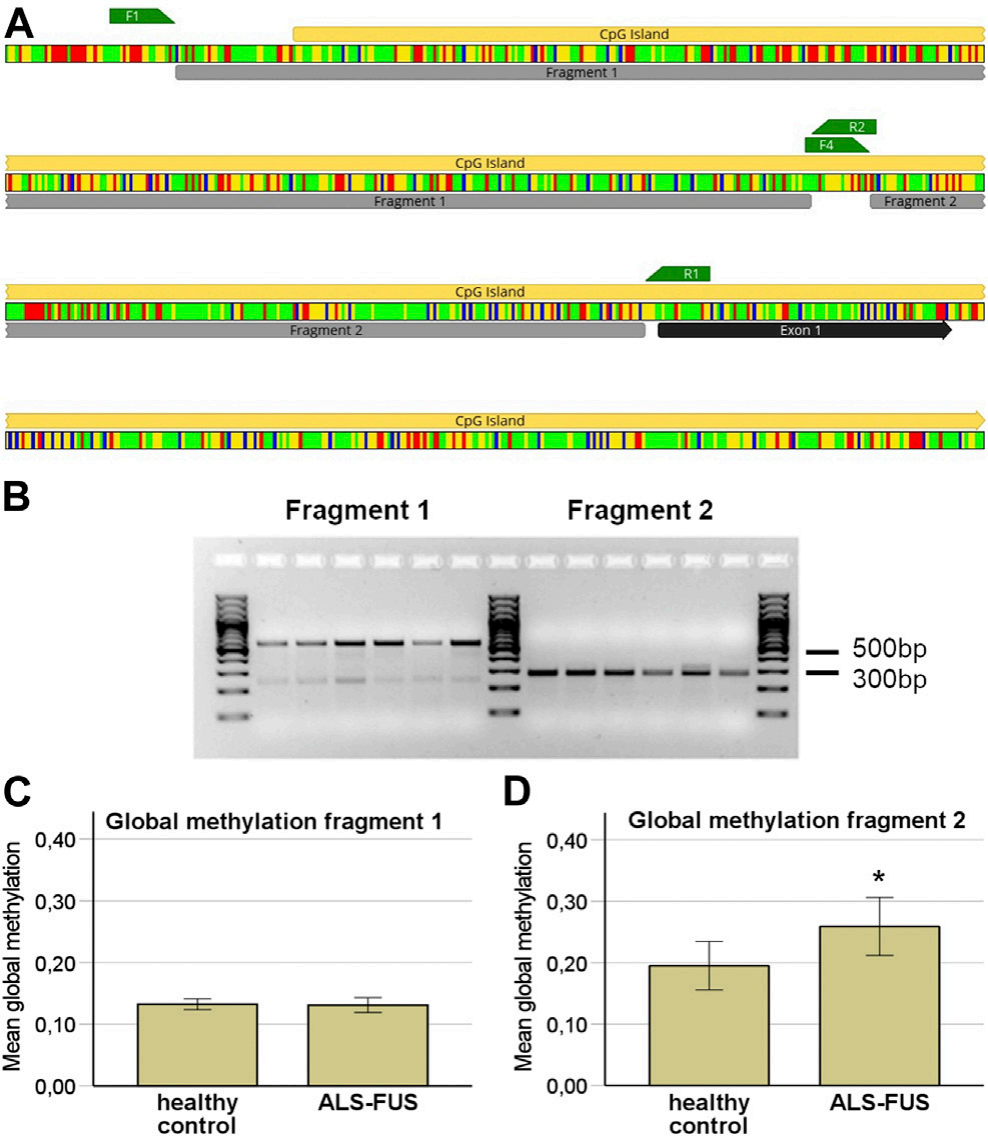
Bisulfite Sequencing of the *FUS* promoter, **(A)** In silico bisulfite modified DNA sequence of the FUS promoter region. Colors represent DNA bases; Adenine: red, Cytosine: blue, Guanine: yellow, Thymine: green. Bisulfite sequencing primers: F1 forward and R2 reversed primer for fragment one and F4 forward primer and R1 reversed primer for fragment 2, **(B)** Gel electrophoresis of the two bisulfite sequencing fragments of the *FUS* promoter, **(C)** Global *FUS* promoter methylation independent of differentiation status showed no significant difference between cell lines in fragment 1, **(D)** ALS cell lines display a significant higher mean global methylation in fragment 2 (independent samples *t*-test; *p* < 0.05, *n* = 3 per condition and *n* = 3 per cell line).

**FIGURE 4:**
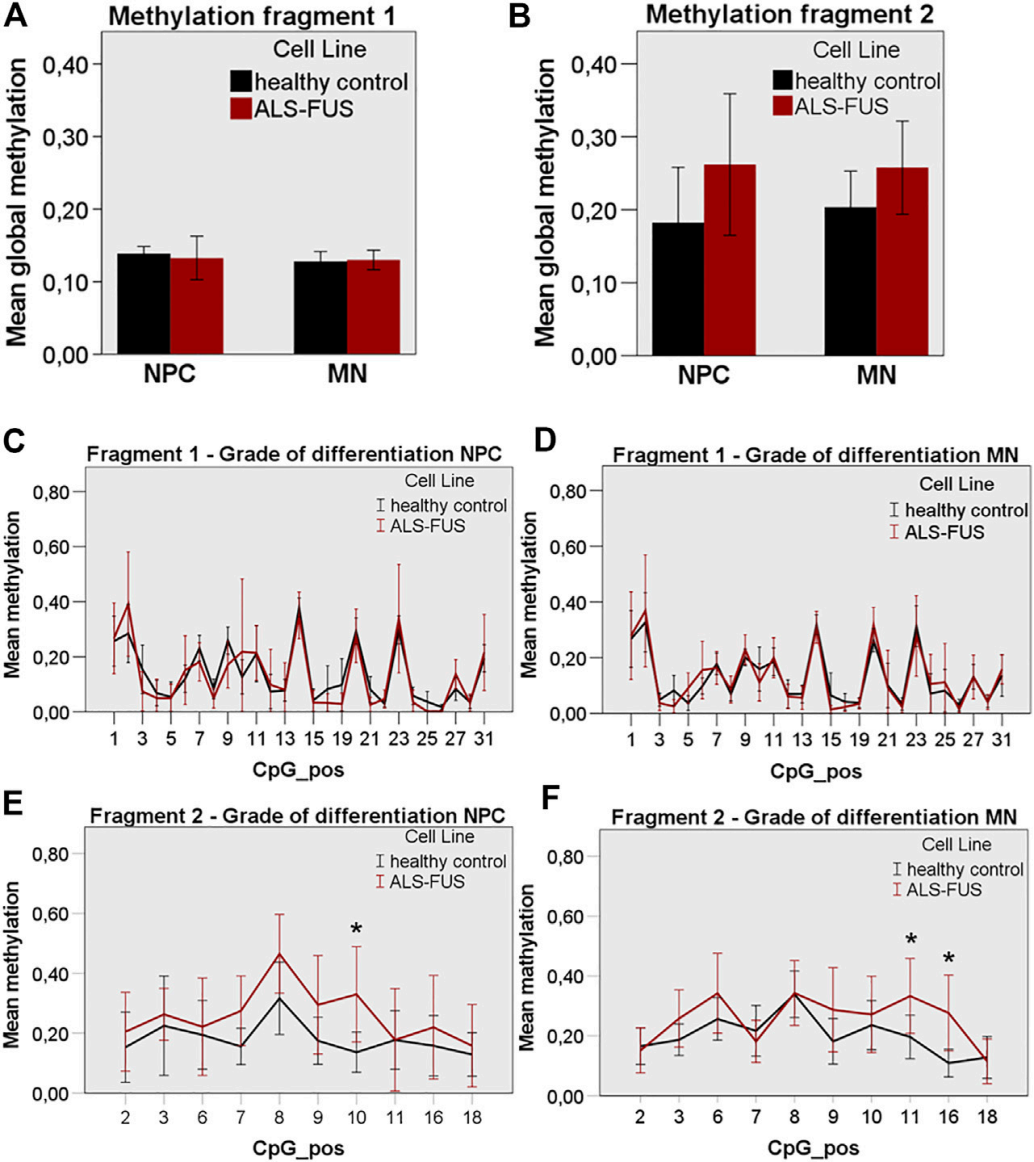
*FUS* promoter methylation in control and ALS cell lines, **(A)** ALS cell lines showed similar global FUS promoter methylation in NPC and MN state compared to control cell lines in fragment 1, **(B)** ALS cell lines showed non-significantly increased global *FUS* promoter hypermethylation in NPC and MN state compared to control cell lines in fragment 2, **(C)** No significant CpG site-specific methylation difference in control and ALS NPCs and, **(D)** MNs in fragment 1, **(E)** Significant CpG site-specific hypermethylation in ALS NPCs at position 10 and, **(F)** in ALS MNs at position 11 and 16 in fragment 2. Statistical inferences were drawn from unpaired *t*-test and illustrated as mean ± SEM. Results were regarded as statistically significant with *p* < 0.05, *n* = 3 per condition and *n* = 3 per cell line.

**FIGURE 5:**
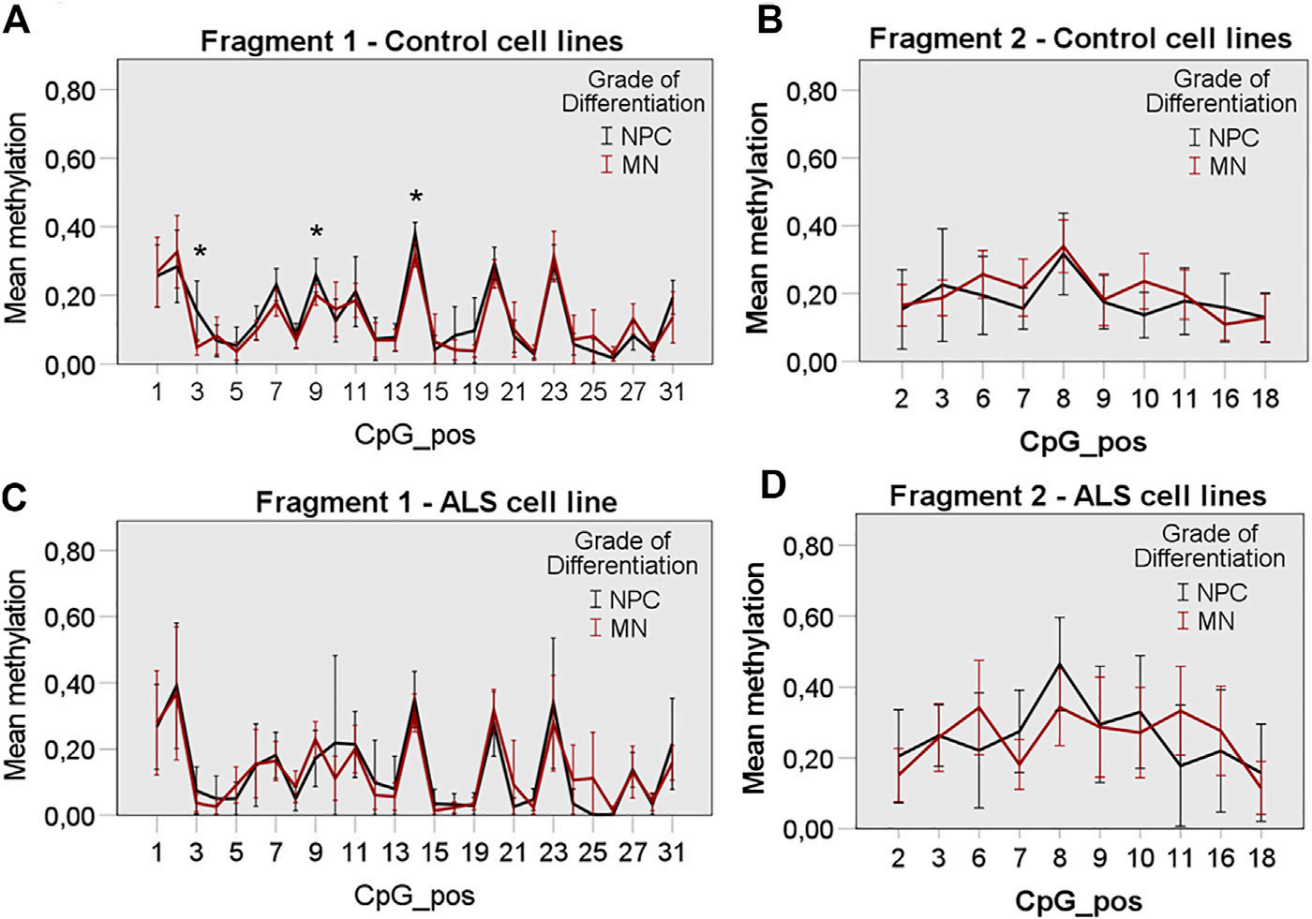
CpG site-specific methylation of the *FUS* promoter during MN differentiation. Control cell lines display a significant methylation change at CpG position 3, 9 and 14 during differentiation in fragment 1, **(A)** but no significant methylation change during differentiation in fragment 2, **(B)**. ALS cell lines display no significant CpG site-specific methylation change in fragment 1, **(C)** and fragment 2, **(D)** during differentiation. Statistical inferences were drawn from unpaired *t*-test and illustrated as mean ± SEM. Results were regarded as statistically significant with *p* < 0.05, *n* = 3 per condition and *n* = 3 per cell line.

**FIGURE 6:**
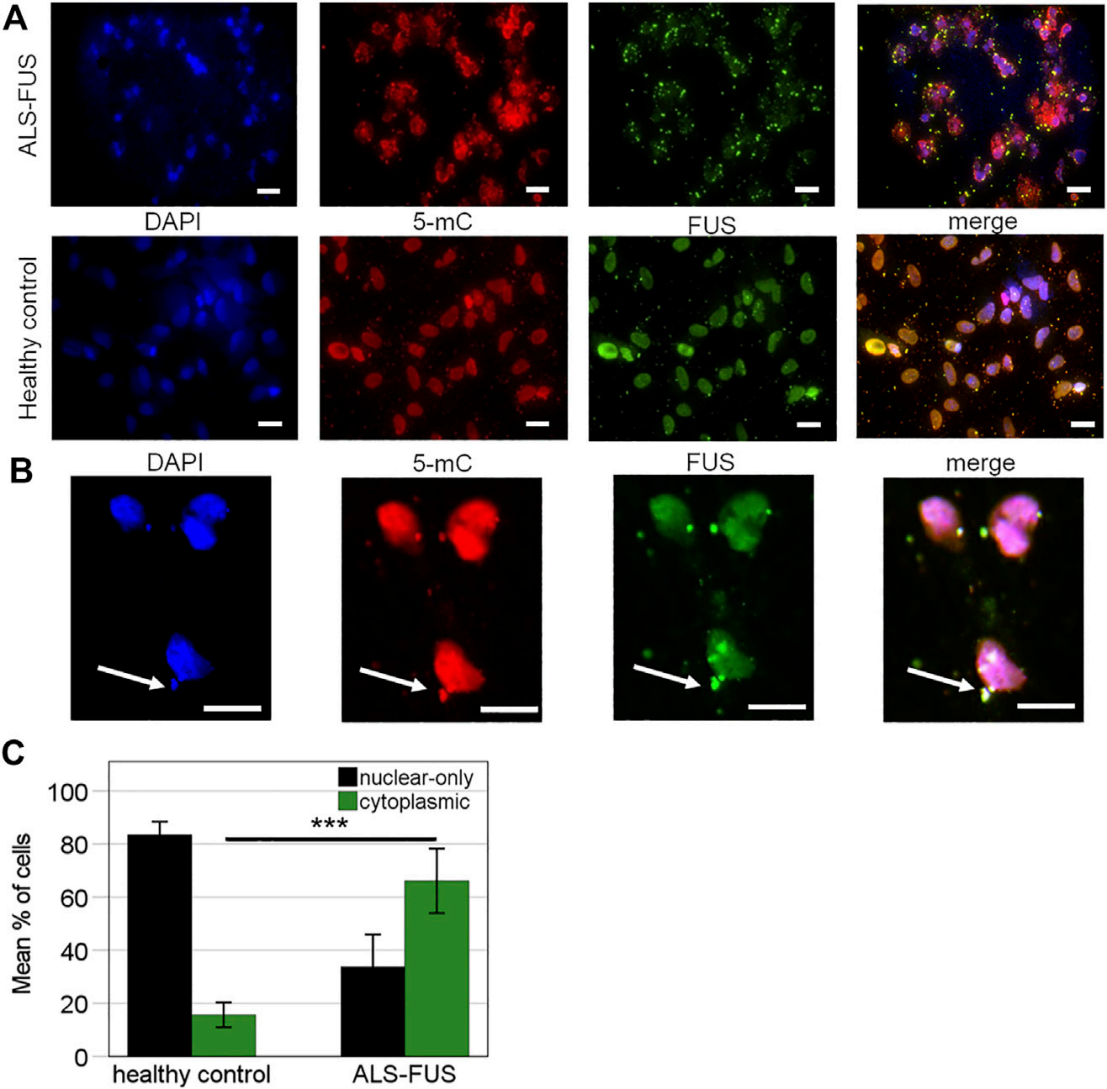
Immunofluorescence of ALS pathology, **(A)** Cytoplasmic retention of mutant *FUS*. *FUS* immunostaining in *FUS* mutant and control MNs. Staining patterns for *FUS* (green), 5mC (red, 5th cytosine methylation) are shown individually and in the merged images together with DAPI-stained nuclei (blue). Scale bars = 20 μm, *n* = 3 per condition and *n* = 3 per cell line, **(B)** ALS motor neurons exhibit cytoplasmic *FUS* aggregates, all of which show perceived proximity with 5-mC (arrows). Scale bar = 20 µm, **(C)** Percentage of cells observed with nuclear-only (dark bars) and any cytoplasmic (green bars) *FUS* staining. Statistical inferences were drawn from unpaired *t*-test and illustrated as mean ± SEM. Results were regarded as statistically significant with ***p < 0.001, *n* = 3 per condition and *n* = 3 per cell line.

We designed two Bisulfite-Sequencing primer pairs for the *FUS* promoter. Bisulfite-Sequencing of a 467 and 211 bp region in a CpG island of the *FUS* promoter was performed capturing quantitative DNA methylation measurements for 35 and 19 individual cytosines (Figure 3A). Bisulfite-Sequencing primers generated two sufficient PCR products (Figure 3B) termed fragment 1 (467 bp) and fragment 2 (211 bp). Mutant *FUS* NPCs and MNs showed no significant change in mean global methylation in fragment 1 (Figure 3C) but significantly higher mean global methylation levels in fragment two compared to control NPCs and MNs (independent samples T-Test *p* < 0.05) (Figure 3D). A subgroup analysis of NPC and MN confirmed no significant difference in fragment 1 (Figure 4A) but a trend towards hypermethylation in mutant *FUS* NPCs and MNs in fragment two clearly remains (Figure 4B). We further analyzed CpG site-specific methylation between control and ALS cell lines and during differentiation. CpG sites in the promoter are numbered as captured on each fragment upstream of the first exon. In fragment one no CpG showed significant site-specific methylation difference in control and mutant *FUS* NPCs and in control and mutant *FUS* MNs (Figures 4C,D). In fragment two CpG site 10 showed significant hypermethylation in mutant *FUS* NPCs compared to control NPCs and CpG site 11 and 16 showed significant hypermethylation in *FUS* mutant MNs compared to control MNs (Figures 4E,F)*.* An in silico transcription factor search revealed factors that can, in relation to other conditions, act both as activator or repressor (Supplementary Figure S3). Further experiments will be necessary to elucidate the role these factors play in the expression of FUS. Analysis of CpG site-specific methylation during differentiation revealed significant hypermethylation of CpG sites 3, 9 and 14 in fragment one in control cell lines (Figure 5A), no CpG site-specific significant difference in control cell lines in fragment 2 (Figure 5B) and no significant difference in *FUS* mutant cell lines in both fragments (Figures 5C,D). In order to visualize potential correlations between aggregate formation as neuropathological hallmark of ALS and methylation differences in mutant *FUS* MNs, we carried out additional immunofluorescence stainings of MNs using 5-mC and *FUS* antibodies. ALS motor neurons exhibited typical pathologic cytoplasmic *FUS* aggregates, which show 5-mC immunoreactivity (Figures 6A,B). ALS MNs showed significantly more cytoplasmic *FUS* aggregates compared to control MNs (independent samples T-Test *p* < 0.05) (Figure 6C). Besides dual staining, single-labeled control stainings confirmed cytoplasmic *FUS* and cytoplasmic 5-mC positive aggregates, supporting co-localisation rather than bleed-through artifacts of fluorescence emission (Supplementary Figure S2).

## Discussion

There is increasing evidence for a role of alterations in DNA methylation in ALS pathogenesis (Martin and Wong, 2013). Even though controversial data have been reported in relation to different ALS causing genes and sporadic ALS cases, the majority of studies come to the conclusion that global DNA methylation is increased in ALS (Chestnut et al., 2011; Tremolizzo et al., 2014). It has, however, not yet been fully elucidated whether DNA methylation changes are associated with distinct ALS causing gene mutations. *FUS* is an ubiquitously expressed protein, localized primarily in the nucleus. It is involved in DNA repair and the regulation of gene transcription, RNA splicing, and export to the cytoplasm. The majority of disease-causing mutations localize at the C-terminal region, resulting in abnormal cytoplasmic retention of *FUS*, which on the one hand impairs its physiological function and on the other hand leads to a toxic gain of function (Kwiatkowski et al., 2009; Vance et al., 2009). Overexpression of mutant *FUS* in cell lines and animal models could induce ALS-like phenotypes (Huang et al., 2011; Markert et al., 2020) but also overexpression of wild-type *FUS* causes progressive motor neuron degeneration (Mitchell et al., 2013).

In the present study, *FUS* mutant motor neurons differentiated from patient-derived iPSCs were used as *in vitro* model of ALS. In prior studies we demonstrated that MNs differentiated by our well-established protocol are healthy and viable via calcium imaging and patch-clamp analysis after 30 days (Naujock et al., 2014). Due to limited availability we did not repeat these experiments for the present study. We observed robust differentiation of human iPSCs into highly enriched MN cultures (>85%) comparable to recent studies using similar differentiation protocols (Fujimori et al., 2018; Tyzack et al., 2019). In line with the literature, *FUS* mutant MNs recapitulated ALS pathology through a decreased motor neuron ratio and an increase in cytoplasmic *FUS* aggregates (Kwiatkowski et al., 2009; Fujimori et al., 2018; Tyzack et al., 2019). Inverse expression of *DNMTs* and *FUS* in mutant *FUS* MNs implies that increased methylation and repressed *FUS* transcription may be associated with ALS pathology. Cytoplasmic *FUS* mislocation and decreased *FUS* expression are both in line with the loss of function and gain of function hypothesis. Cytoplasmic *FUS* mislocation results in loss of nuclear function and at the same time gain of function through toxic aggregates. Decreased *FUS* mRNA expression compared to healthy controls also may be associated with primary loss of nuclear function and possible gain of function when assuming disturbed autoregulation. We assume that both mechanisms may partly be driven by altered methylation.

Only few studies have investigated *FUS* expression itself in mutant *FUS* ALS models and have come to different conclusions. Sabatelli et al. reported significant overexpression of three rare variants with so far uncertain significance (c.∗59 G.A, c.∗108 C.T and c.∗110 G.A) in fibroblasts from three sporadic ALS patients but no significant difference in one R521C mutant patient (Sabatelli et al., 2013). De Santis et al. (2017) found no significant difference in *FUS* expression by gene editing with introduction of P525L mutation and wild type *FUS* iPSC-derived MNs. These results are in contrast to ours but one has to take into account differences in mutations analyzed, methods applied and cell models. There is only one study to our knowledge so far regarding the R521L *FUS* mutation. Dash et al. (2020) found mutant R521L *FUS* not to be differentially expressed in iPSC-derived MNs from ALS patients in a microarray based study.

Whereas some studies suggest that *DNMT1* and *DNMT3a* are relatively stably expressed during neurogenesis together with stable global 5-mC levels (Wang et al., 2016), other studies showed downregulation of *DNMT3b* and upregulation of *DNMT3a* expression during MN differentiation and showed that loss of *DNMT3a*, but not *DNMT3b*, impairs the ability of embryonic stem cells (ESCs) to efficiently generate MNs (Ziller et al., 2018). In line with these results, Wu et al. (2010) further demonstrated that *DNMT3a* deletion impairs neuronal differentiation and that *DNMT3a* is expressed in diverse regions of the postnatal brain including subependymal/subventricular zones (SEZ/SVZ) of the forebrain and the hippocampal dentate gyrus emphasizing its role in neurogenesis. In contrast, overexpression of *DNMT1* and *DNMT3a* induces neurodegeneration in primary murine neurons, which can be rescued by inhibition of *DNMTs* (Chestnut et al., 2011; Martin and Wong, 2013). On the one hand, *DNMT3a* expression is crucial for normal MN development and on the other hand higher expression levels lead to neurodegeneration. Our study is in line with both of these findings: we found higher *DNMT1*, *DNMT2* and *DNMT3a* mRNA expression in MNs compared to NPCs, but also higher expression levels of *DNMT1*, *DNMT2* and *DNMT3a* in mutant *FUS* MNs compared to control MNs. These results support a role of *DNMTs* in motor neuron development and in addition a potential pathogenic impact in ALS, emphasizing the importance of balanced *DNMT* expression.

While alterations of global DNA methylation have been verified in ALS, the role of site-specific changes still needs to be clarified. The majority of methylation analyses in fALS so far have examined global methylation, focusing on *SOD1* and more recently C9orf72 mutations. Very few studies addressed promoter or site-specific methylation changes. Global DNA methylation in fALS cellular models revealed an increase in 5-mC in cells transduced by mutant *SOD1*, but no significant alterations were observed in cells transduced by wild type (WT) or pathological mutant *FUS* or *TDP-43* indicating that different ALS-causing genes contribute to global epigenome alteration in distinct ways (Masala et al., 2018). Coppede et al. (2018) also confirmed higher global methylation in blood from *SOD1* patients and even a positive correlation with disease duration but the *SOD1* gene promoter was hypomethylated in all patients. Accordingly, promoter specific methylation analysis of *SOD1* in sporadic ALS cases revealed *SOD1* promoter hypomethylation (Oates and Pamphlett, 2007). As mentioned earlier, a number of recent studies reported promoter hypermethylation in *C9orf72* patients corresponding with reduced *C9orf72* expression (Xi et al., 2013; Liu et al., 2014; Russ et al., 2015; Gijselinck et al., 2016; Shi et al., 2018). Xi et al. (2013) reported hypermethylation of 26 CpG sites in a CpG island at the 5′ end of the repeat expansion and reduced *C9orf72* expression in *C9orf72* expansion carriers. Interestingly Liu et al. (2014) describe that expression of mutant *C9orf72* is repressed by promoter methylation and that this results in reduced pathologic accumulation of repeat-containing RNA suggesting that DNA methylation protects cells from neurodegeneration and that mutant *C9orf72* is associated with a toxic gain of function. Shi et al. further demonstrated that reduced *C9orf72* activity disrupts lysosomal biogenesis in motor neurons. These studies underline the counterplay of gain- and loss-of-function mechanisms leading to neurodegeneration. In order to analyze if *FUS* repression is due to promoter hypermethylation, we performed bisulfite-sequencing*.* Even though the detection limit of targeted bisulfite sequencing at each CpG site is about 10–20% compared to e.g., bisulfite pyrosequencing with about 5%, it remains a satisfactory and reliable method among the variety of new methods for methylation analysis with single CpG resolution especially when targeting sequences longer than 100 bp (Mikeska et al., 2010). Our results support an inverse correlation of *FUS* expression and proximal *FUS* promoter methylation in ALS cell lines. CpG site-specific methylation analysis revealed significant differences in three CpG sites in mutant *FUS* cells which may be functionally relevant for *FUS* pathology. We further detected significant differences in methylation of three CpG sites during differentiation in control cell lines, which may be functionally relevant for MN differentiation. Despite of lower *FUS* expression levels, mutant *FUS* MNs exhibit significantly more pathologic cytoplasmic *FUS* aggregates. One could postulate that *FUS* methylation and repression represents a protective mechanism to counteract cytoplasmic aggregate formation. This has similarly been postulated regarding mutant *C9orf72* ALS (Liu et al., 2014). Van Blitterswijk et al. (2013) applied RNA-Sequencing in cells expressing mutant *FUS* transduced HEK-293T cells and compared transcription profiles with cells overexpressing wild-type *FUS* and knock-down *FUS* cell lines. They came to the conclusion that *FUS* mutants resemble wild-type *FUS* overexpressing cells rather than knock-down *FUS* cell lines and therefore postulate that *FUS* mutations do not contribute to ALS pathogenesis through a loss-of-function mechanism. Interestingly, cells carrying *FUS* mutations had lower *FUS* protein levels than wild-type cells suggesting post-transcriptional regulation mechanisms. Nevertheless our data cannot prove a causal relationship between FUS repression and promoter methylation by DNMT upregulation. Alternatively, transcriptional and post-transcriptional repression could be mediated by small RNAs. Whereas microRNAs (miRNAs) and small interfering RNAs (siRNAs) can lead to mRNA degradation directly, PIWI-interacting RNAs (piRNAs) can additionally indirectly repress transcription by causing histone modification and DNA methylation (Huang and Wong, 2021). If small RNAs directly induce repressive epigenetic marks or indirectly by interacting with DNMTs is subject of current research. Independent of biological function small RNAs recently emerged as a powerful gene silencing tool in research and DNMT targeted siRNAs could be applied in further studies for proof of concept if the observed alterations in FUS expression are caused by promoter methylation due to altered DNMT expression.

One post-translational mechanism previously studied in ALS is protein methylation, more precisely arginine methylation, which contributes to mislocation of mutant *FUS* proteins (Dormann et al., 2012). Arginine methylation of the NLS domain of *FUS* modulates Transportin binding to *FUS* and hence its nuclear import (Dormann et al., 2012). *FUS* aggregates in ALS postmortem tissue and iPSC-derived MNs co-stain for arginine methylation and inhibition of arginine methylation is known to restore *FUS* mislocation in iPSC-derived MNs (Japtok et al., 2015; Naumann et al., 2018). The antibody used for detection targets an epitope in 31 amino acids from the C-terminal region of *FUS* with all arginines methylated. In our study, in contrast, we used an anti-5-mc antibody targeting methylated cytosines in DNA and RNA and observed co-staining of cytoplasmic *FUS* aggregates and 5-mC. In line with our results, Chestnut et al. described increased 5-mC immunoreactivity in motor neurons undergoing apoptosis (Chestnut et al., 2011). Accordingly, as we show perceived proximity of 5-mC with cytoplasmic *FUS* aggregates, we hypothesize that 5-mC contributes to the pathological *FUS* recruitment and stress granule aggregation in ALS, whereas arginine methylation contributes to pathological mislocation (Naumann et al., 2018) presuming synergistic impact. Inhibition of 5-mC methylation could potentially restore aggregation of mutant *FUS* and its expression to wild-type levels.

It has recently been discovered that 5-mC contributes to posttranslational modifications in coding and non-coding RNA (Zhao et al., 2017) and that RNA methylation by *DNMT2* protects transfer RNAs against stress-induced cleavage (Schaefer et al., 2010). Stress granules contain high amounts of RNA, including transfer-RNA (t-RNA) (Kedersha et al., 2002) and mis-processing of RNA-binding proteins such as *FUS* and stress granules has been shown to be pathogenic in ALS suggesting (Zhang et al., 2020) that *DNMT2*-mediated t-RNA modification plays a role in the cellular stress response (Bohnsack et al., 2019). Besides t-RNA, *DNMT2* and methylated cytosines in mRNA promote the nuclear export of RNAs (Yang et al., 2017). Our results are in line with these studies since our anti-5-mc antibody does not differentiate between cytosine methylation in DNA, tRNA or mRNA.

Our results therefore indicate that up-regulation of DNMT1 and DNMT3a could represent an attempt to induce downregulation of FUS in order to minimize cytoplasmic aggregates and that upregulation of DNMT2, followed by increased t-RNA methylation may counteract t-RNA cleavage and stress granule formation.

First studies on modification of DNA methylation in ALS have been promising. Oh et al. (2016) showed that human bone marrow mesenchymal stromal cells (MSCs) isolated from ALS patients have functionally decreased differentiation potential and excessively express *DNMTs*. Functional restoration of ALS patient-derived MSCs was achieved using the *DNMT1* inhibitor RG108. Fujii et al. (2016) demonstrated that treatment with the global methyltransferase inhibitor adenosine dialdehyde could alleviate cytoplasmic mislocation and aggregation of mutant *FUS*. These studies underline the potential of epigenetic modulation as therapy strategy. Beside *DNMT* inhibitors, Histone deacetylase inhibitors (HDACi) showed functional restoration of ALS patient-derived MNs by reversing axonal transport defects (Guo et al., 2017).

As mentioned earlier histone modification is an epigenetic mechanism modeling chromatin state. HDACis interact with DNMTis and can act synergistically to reactivate silenced genes in disease model (Yang et al., 2001). HDACi decreases *DNMT1* expression and therefore synergistically results in hypomethylation (Arzenani et al., 2011). Our results are in line with research addressing HDAC function in ALS and further emphasize interaction of epigenetic factors.

In summary, we have successfully differentiated MNs from ALS patient-derived iPSCs carrying a *FUS* mutation. These MNs showed typical ALS pathology with cytoplasmic *FUS* aggregates. *FUS* expression was reduced in ALS MNs in association with higher expression of DMNT 1, 2 and 3a and higher proximal *FUS* promoter methylation. *FUS* aggregates showed 5mC immunoreactivity supporting a role of DNA and RNA methylation in ALS pathology. Further studies with more replicates and isogenic controls as well as analyses of FUS promotor methylation in post mortem tissue from both patients with sALS and fALS will be necessary to confirm our findings in order to investigate possibilities of epigenetic modification as therapeutic strategy.

## Data Availability

Datasets are available on request to the corresponding author.
