# Supplementary material for: Methylation and Expression of Mutant FUS in Motor Neurons Differentiated From Induced Pluripotent Stem Cells From ALS Patients
Source: Front Cell Dev Biol. 2021 Nov 19;9:774751. doi: 10.3389/fcell.2021.774751 (PMC8640347; doi:10.3389/fcell.2021.774751)
Supplement: Supplementary file 1 [file DataSheet1.PDF]

# Supplementary Table 1

| iPSC line | NPC line | gender | Year of birth | Mutation |
|---|---|---|---|---|
| iPSC_FUS_1 | NPC_FUS_1 | female | 1952 | FUS R521C |
| iPSC_FUS_2 | NPC_FUS_2 | female | 1944 | FUS R521L |
| iPSC_FUS_3 | NPC_FUS_3 | female | 1952 | FUS R521C |
| iPSC_CON_1 | NPC_CON_1 | female | 1959 | - |
| iPSC_CON_2 | NPC_CON_2 | male | 1949 | - |
| iPSC_CON_3 | NPC_CON_3 | female | 1963 | - |

**Table S1**. Induced pluripotent stem cell (iPSC), neural progenitor cell (NPC) lines and patient characteristics.

## Supplementary Table 2

| gene | forward primer | reversed primer | amplicon size |
|---|---|---|---|
| GAPDH | GAA GGT GAA GGT CGG AGT C | GAA GAT GGT GAT GGG ATT TC | 226 bp |
| PPIA | GCC GAG GAA AAC CGT GTA CT | TGT CTG CAA ACA GCT CAA AGG | 109 bp |
| TUJ1 | AGT GAT GAG CAT GGC ATC GAC CC | GGC ACG TAC TTG TGA GAA GAG GC | 110 bp |
| MAP2 | CAG GCA AAG GAC AAA GTC TCT GAC G | CGC CGA GGA GGG AGA ATG GAG G | 92 bp |
| ISLET1 | AAG GAC CAA CTG GTA GAG ATG ACG G | GCT GAT CTA TGT CAC TCT GCA AGG C | 286 bp |
| SMI32 | ACC TGC TCA ATG TCA AGA TGG CTC | AAA GCC AAT CCG ACA CTC TTC ACC | 86 bp |
| FUS | CAA GGT CTC ATT TGC TAC TCG CCG | TCA CAG GTG GGA TTA GGA CAC TTC C | 210 bp |
| DNMT1 | CCA GGC AAA CCA CCA TCA CAT CTC | AGC GGT CTA GCA ACT CGT TCT CTG | 166 bp |
| DNMT2 | TCC AGA GAA CGA GTT GCT AGA CCG | GCT CCT CCT TCA GTT TCT GTT TGG G | 160 bp |
| DNMT3a | TGA CCC TCC AAA GGT TTA CCC ACC | GGA TAT GCT TCT GTG TGA CGC TGC | 236 bp |
| DNMT3b | CCA GAG AAC AAG ACT CGA AGA CGC | TTT CCT GCC ACA AGA CAA ACA GCC | 201 bp |

**Table S2**. Primer sequences used for expression analyses by quantitative real-time polymerase chain reaction.

# Supplementary Figure 1

A

B

C

D

E

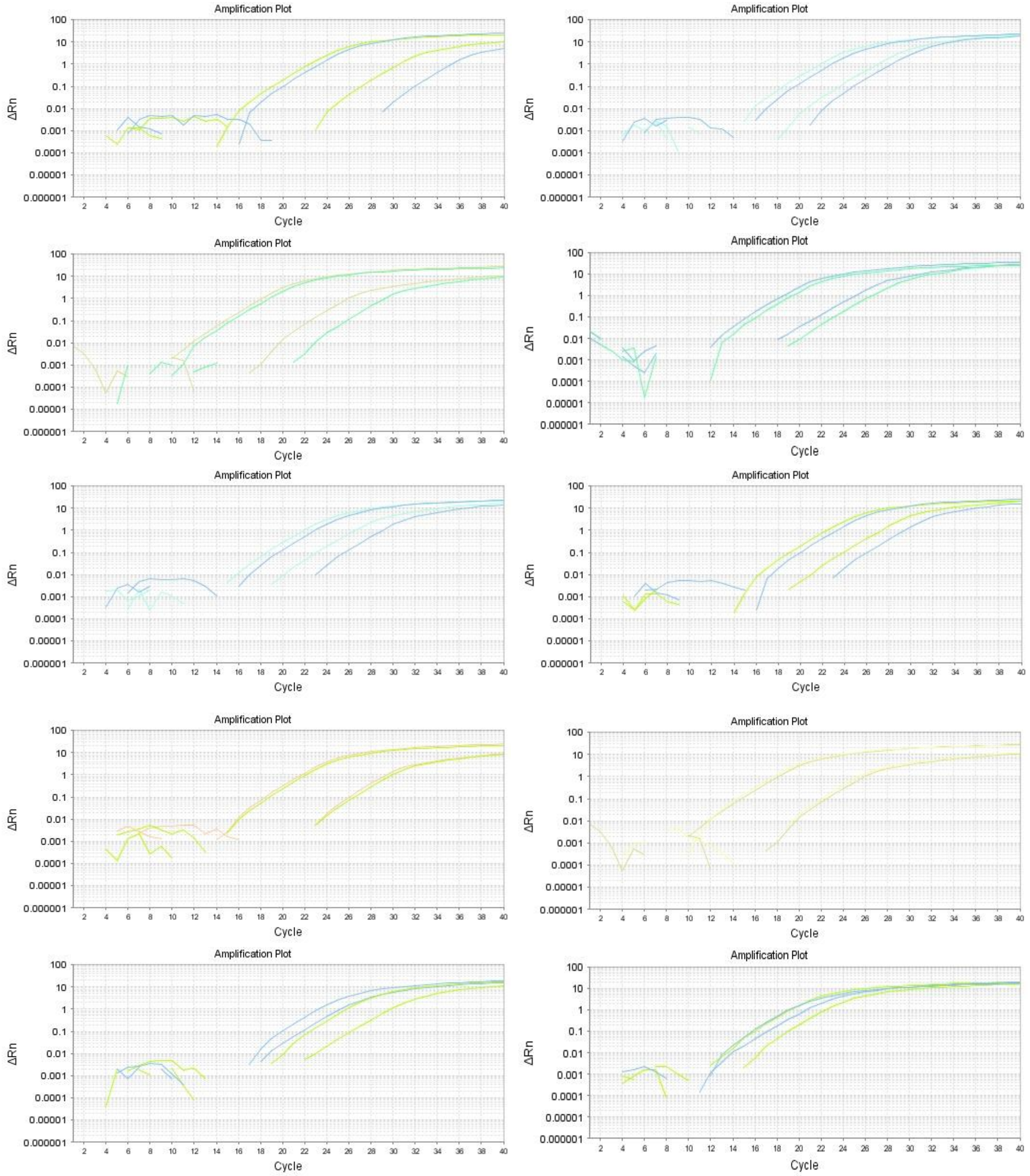


F

G

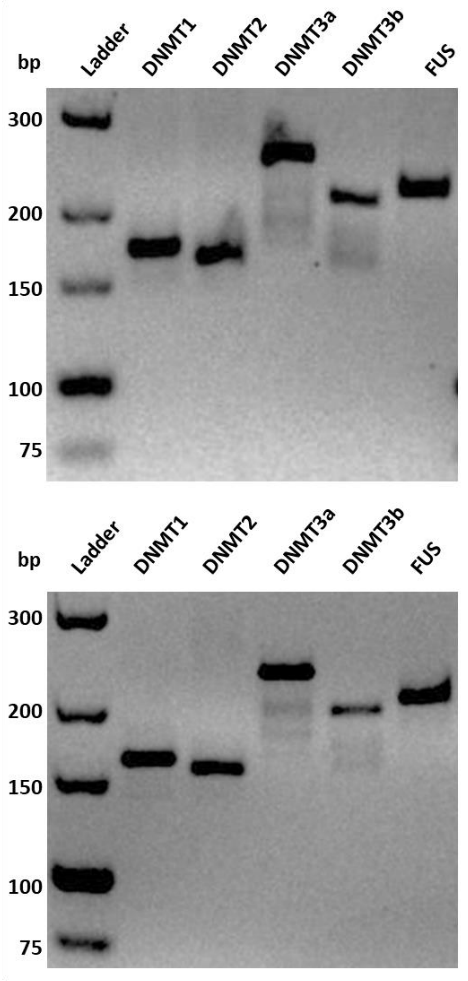


**Figure S1. Examples of real time PCR amplification plots of DNMTs and FUS in control and ALS MNs. Each image shows the reference (GADPH or PPIA) and target gene amplification of one control and one ALS MN sample**

A) DNMT1
   left panel blue: control, green: ALS
   right panel blue: control, light blue: ALS
B) DNMT2
   left panel green: control, orange: ALS
   right panel green: control, blue: ALS
C) DNMT3a
   left panel blue: control, light blue: ALS
   right panel blue: control, light green: ALS
D) DNMT3b
   left panel brown: control, light green: ALS
   right panel yellow: control, brown: ALS
E) FUS
   left panel blue: control, light green: ALS
   right panel blue: control, light green: ALS

Amplicon verification. Agarose gel electrophoresis showing PCR products of the expected size for each gene in control and ALS MNs.

F) Control MNs
G) ALS MNs

# Supplementary Figure 2

A B

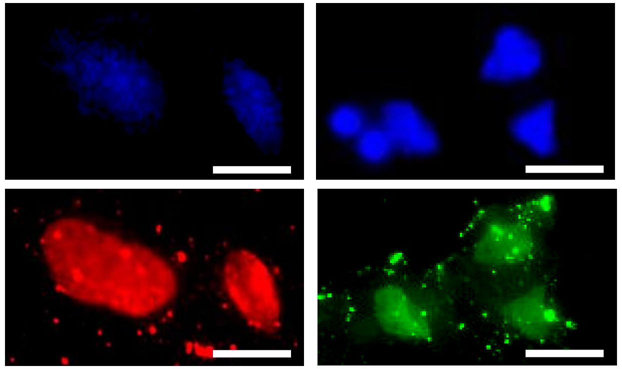

**Figure S2. Immunofluoresence of ALS pathology. Single control stainings A)** Cytoplasmatic mislocation of 5mC. Single staining with DAPI and 5mC, accordingly Alexa Fluor 555. **B)** Cytoplasmatic mislocation of FUS. Single staining with DAPI and FUS, accordingly Alexa Fluor 488. Scale bar = 20µm

## Supplementary Figure 3

Transcription factor prediction for significant CpGs from Fragment 2 (proximal promoter region)

| TF | Tissue CpG | Sequence | Literature | Uniprot link | Rating (+,-,+/-) | Consensus |
|---|---|---|---|---|---|---|
| **NRF1** | 10,11 | GGTG**C**G**C**GCCTG | https://pubmed.ncbi.nlm.nih.gov/9421508 | http://www.uniprot.org/uniprot/ZBT14_HUMAN | +/- | 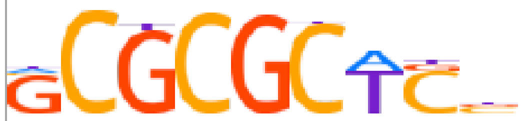 |
| **ZBTB14** | 10,11 | GGTG**C**G**C**GCCTG | https://pubmed.ncbi.nlm.nih.gov/17714511 | https://www.uniprot.org/uniprot/Q14494 | +/- | 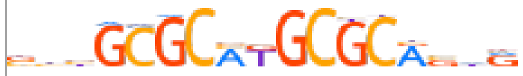 |
| **TCFL5** | 10,11 | GGTG**C**G**C**GCCTG | https://pubmed.ncbi.nlm.nih.gov/9763657 | https://www.uniprot.org/uniprot/Q9UL49 | +/- | 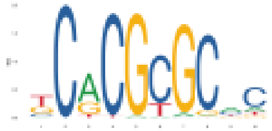 |
| **EGR1** | 10,11 | GGTG**C**G**C**GCCTG | https://pubmed.ncbi.nlm.nih.gov/20121949 | http://www.uniprot.org/uniprot/P18146 | + | 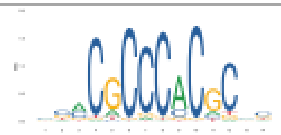 |
| **SP1** | 16 | GCGG**C**GGAGC | **https://pubmed.ncbi.nlm.nih.gov/20091743** | https://www.uniprot.org/uniprot/P08047 | +/- | 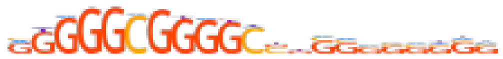 |
| **SP3** | 16 | GCGG**C**GGAGC | https://pubmed.ncbi.nlm.nih.gov/15494207 | https://www.uniprot.org/uniprot/Q02447 | +/- | 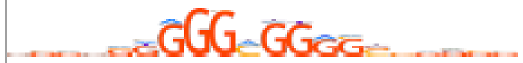 |
| **KLF12** | 16 | GCGG**C**GGAGC | | http://www.uniprot.org/uniprot/KLF12_HUMAN | - | 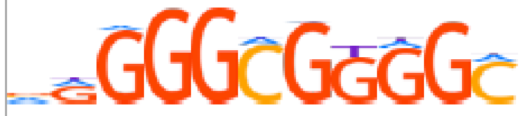 |
